# Chronic Rhinosinusitis with Nasal Polyps: T-Cell Dysregulation and Regulatory T-Cell Dysfunction

**DOI:** 10.1007/s40136-026-00556-w

**Published:** 2026-10-29

**Authors:** Ebrahim Farashi, Tolani F. Olonisakin, Joshua M. Levy, Nyall R. London

**Affiliations:** 1 Sinonasal and Skull Base Tumor Section, Surgical Oncology Program (SOP), Center for Cancer Research (CCR), National Cancer Institute (NCI), National Institutes of Health (NIH), Johns Hopkins University School of Medicine, Bethesda, MD USA; 2 Department of Otolaryngology–Head and Neck Surgery, Johns Hopkins University School of Medicine, Baltimore, MD USA; 3 Sinonasal and Olfaction Program, National Institute on Deafness and Other Communication Disorders (NIDCD), National Institutes of Health, Bethesda, MD USA; 4 Department of Otolaryngology–Head and Neck Surgery, Emory University School of Medicine, Emory University, Atlanta, GA USA; 5 Department of Neurosurgery, Johns Hopkins University School of Medicine, Baltimore, MD USA; 6 Department of Oncology, Johns Hopkins University School of Medicine, Baltimore, MD USA

**Keywords:** Chronic rhinosinusitis with nasal polyps, T helper cells, Regulatory T-cells, Type 2 inflammation, Biologics

## Abstract

**Purpose of Review:**

Chronic rhinosinusitis with nasal polyps (CRSwNP) is a heterogeneous inflammatory disorder driven by dysregulated innate and adaptive immune responses. This review summarizes current evidence on the roles of T-helper cell subsets and regulatory T-cells (T_regs_) in CRSwNP pathogenesis and discusses their implications for disease persistence and targeted therapy.

**Recent Findings:**

Beyond classical Th2-driven inflammation, recent studies demonstrate important contributions from Th1, Th9, Th17, Th22, and CD8⁺ effector-memory/tissue-resident T-cells to epithelial injury, tissue remodeling, and disease recurrence. Reduced FOXP3⁺ T_reg_ frequency and impaired function, together with alterations in IL-2, TGF-β, and IL-10 signaling, may contribute to persistent immune activation and inflammatory heterogeneity. Advances in biologic therapies targeting type 2 inflammation have substantially improved outcomes in selected patients.

**Summary:**

T-cell dysregulation is central to the immunopathogenesis of CRSwNP. A better understanding of immune endotypes, T-cell plasticity, and regulatory pathways may facilitate biomarker-guided patient stratification and support the development of more precise immunomodulatory therapies.

## Introduction

Chronic rhinosinusitis with nasal polyps (CRSwNP) is a subtype of chronic rhinosinusitis (CRS) characterized by benign inflammatory polyps arising from the chronically inflamed sinonasal mucosa [1, 2]. CRS affects approximately 5–12% of the adult population, representing a substantial global health burden because of its impact on quality of life and healthcare utilization [3, 4]. In the United States, the prevalence of CRSwNP is estimated to be 1–4% among adults and it accounts for approximately 25–30% of all CRS cases [5, 6]. CRS is now recognized as a biologically heterogeneous disease with distinct inflammatory endotypes defined by molecular and cytokine signatures. Endotype-based classifications recognize three major inflammatory patterns [7]. Type 1 inflammation is primarily characterized by IFN-γ–mediated immune responses, type 2 by IL-4, IL-5, and IL-13 with eosinophilic inflammation, and type 3 by IL-17 and IL-22, which is mainly associated with neutrophilic inflammation. Biomarker-based studies have shown that clinical phenotypes and inflammatory endotypes do not always correspond, with frequent overlap and mixed immune signatures [8]. Multicenter analyses have identified substantial geographic variation in T-helper cytokine patterns, with type 2 inflammation predominating in Western cohorts and more heterogeneous or Th1/Th17-associated profiles reported in several Asian populations [9]. This endotypic and geographic diversity should be considered when interpreting immunologic variability and T-cell dysregulation in CRSwNP. Despite the availability of treatments such as topical corticosteroids and surgical intervention, obtaining long-term control of NPs remains challenging, as recurrence is common even after resection [10, 11]. Persistent inflammation in CRSwNP results from complex interactions between innate and adaptive immune responses rather than a single inflammatory pathway [12]. This review focuses on the role of T-cell dysregulation, particularly regulatory T-cell dysfunction, in the pathogenesis of CRSwNP and its potential clinical implications.

## T-cells in Nasal Polyps and their Relation to Disease Pathogenesis

NPs can exhibit tertiary lymphoid tissue-like features as they are enriched in T and B cells that contribute to local immune responses [13]. Activated T-cells differentiate into functionally distinct subsets with diverse roles in CRSwNP pathogenesis. Signals derived from dendritic cells, innate lymphoid cells, and the epithelium regulate T-cell differentiation. The major T-cell subsets involved in CRSwNP, together with their principal cytokines, clinical implications, and therapeutic relevance, are summarized in Table 1.

**Table 1:** T-cell subsets in chronic rhinosinusitis with nasal polyps: key cytokines and medicators, immunologic roles, clinical implications, and therapeutic relevance

T-cell subset	Key cytokines/mediators	Role in CRSwNP	Endotype heterogeneity	Therapeutic relevance
Th1	IL-2, IFN-γ	Cell-mediated immunity; type 1 inflammation	Disease heterogeneity; cell-mediated immunity	Emerging immunomodulatory target
Th2	IL-4, IL-5, IL-13	Eosinophilic inflammation	Severe disease, asthma, recurrence	Approved biologics
Th17	IL-17, IL-21, IL-22	Neutrophilic inflammation	Treatment resistance	Emerging target
Th9	IL-9	Amplification of inflammation	Emerging pathogenic pathway	Experimental
Th22	IL-22	Epithelial homeostasis and repair	Epithelial dysfunction	Emerging target
CD8⁺ T cells	GZMK, IFN-γ	Tissue injury and recurrence	Postsurgical recurrence	Emerging target
FOXP3⁺ T_regs_	IL-10, TGF-β, IL-35	Immune tolerance	Persistent disease; immune dysregulation	Emerging immunomodulatory target

CRSwNP, chronic rhinosinusitis with nasal polyps; T_regs_, regulatory T cells; IL, interleukin; IFN-γ, interferon-γ; GZMK, granzyme K

### CD8 + T-cells

The sinus mucosa in CRSwNP is characterized by a marked accumulation of both CD8⁺ and CD4⁺ T-cells, with CD8⁺ T-cells mainly demonstrating an effector-memory phenotype. These findings suggest that CD8⁺ T-cells may actively contribute to disease pathogenesis rather than merely reflecting ongoing inflammation [14]. Supporting this, another study comparing lymphocytes isolated from NPs with those from peripheral blood showed a relative decrease in CD4⁺ T-cells and an increased proportion of CD8⁺ T-cells in polyp-derived samples. In both T-cell populations, there was a consistent shift from naïve cells in the circulation toward effector-memory and terminally differentiated subsets within the polyp tissue [15]. More recently, a distinct pathogenic subset of granzyme K (GZMK⁺) effector-memory CD8⁺ T-cells has been identified in recurrent NPs. These cells exhibit clonal expansion and tissue-resident persistence, indicating a sustained pathogenic role. Functionally, GZMK⁺ CD8⁺ T-cells engage complement-associated inflammatory pathways, promote local inflammation, and contribute to polyp recurrence [16, 17]. Additional studies have demonstrated increased activation and cytotoxic activity of CD8⁺ T-cells, with their localization at epithelial–stromal interfaces suggesting direct involvement in mucosal inflammation and tissue remodeling [18–20]. These findings indicate that CD8⁺ T-cells play a broader pathogenic role in persistent inflammation, tissue remodeling, and disease recurrence in CRSwNP.

### Th1 Cells

Th1 cells trigger cell-mediated immune responses [21]. Th1 responses promote the activation of CD8⁺ T-cells, macrophages, NK cells, and immunoglobulin G–producing B cells. The key Th1 transcription factors are the signal transducer and activator of transcription 4 and T-box transcription factor TBX21 (T-bet). Th1 cells are triggered by the polarizing cytokine interleukin (IL)-12, and their effector cytokines are IL-2 and interferon-gamma (IFN-γ). Th1 cells also produce tumor necrosis factor-alpha (TNF-α), which further amplifies inflammatory responses [22, 23]. CRS without nasal polyps (CRSsNP) has historically been associated with a Th1-skewed immune response. However, recent biomarker-based studies have revealed marked immunologic heterogeneity within CRSsNP, including mixed Th1/Th17 profiles and immune signatures that partially overlap with healthy control tissue [24, 25]. Although the overall contribution of Th1 cells to NP development remains uncertain, the cytokines they produce may contribute to anti-polyp immunity and counteract Th2-driven eosinophilic inflammation. Among Th1-associated cytokines, IL-2 is of particular interest because of its essential role in the expansion, maintenance, and suppressive function of T_regs_, which are critical for immune tolerance and inflammation control. Further studies are required to determine whether Th1 activation directly contributes to polyp suppression or plays a modulatory role.

### Th2 Cells

Th2 cells induce type 2 immune responses. These cells secrete IL-4, IL-5, and IL-13, promote humoral immunity through B-cell activation, mediate host defense against helminths, facilitate tissue repair, and contribute to chronic inflammatory diseases such as asthma and allergic disorders. Cytokines secreted by Th2 cells stimulate immunoglobulin E (IgE) production and recruit eosinophils to tissues, leading to tissue inflammation and remodeling [26, 27]. Th2 cells play a major role in CRSwNP, particularly in the maintenance of inflammation and recruitment of eosinophils and other immune cells. Cytokines secreted by Th2 cells are associated with eosinophilic infiltration in NPs in the United States and Europe [28, 29] and these cytokines have a central role in the pathogenesis of NP formation in Western populations. However, other Th subsets and immune cells also contribute to CRSwNP, resulting in a complex and heterogeneous inflammatory profile. A better understanding of the interactions among T-cell-derived cytokines, epithelial cells, fibroblasts, and other immune cells may help identify additional therapeutic targets.

In a recent single-cell study of NPs from patients with CRSwNP, distinct populations of CD4⁺ cytotoxic T-cells and IL-10⁺CD109⁺ Th2 cells with potential regulatory functions were identified [30]. This finding supports the concept that Th2-dominant inflammation in CRSwNP coexists with phenotypically and functionally diverse CD4⁺ T-cell populations. Clinically, type 2 inflammation frequently coexists with asthma and aspirin-exacerbated respiratory disease, a phenotype associated with greater disease severity and increased disease burden [31].

### Th17 Cells

Th17 cells represent an important pro-inflammatory T-helper subset implicated in the pathogenesis of CRSwNP. A study demonstrated a significant increase in Th17 cells within NP tissue, accompanied by elevated production of Th17-associated cytokines, including IL-17A , IL-17F, IL-21, and IL-22, compared with CRSsNP and healthy controls [32]. These Th17 cytokines contribute to mucosal inflammation by promoting neutrophil recruitment, epithelial barrier disruption, and sustained inflammatory signaling within the sinonasal microenvironment [32, 33]. Th17-associated immune signatures appear to be endotype- and population-dependent. Western CRSwNP cohorts often show mixed Th2/Th17 inflammatory patterns, while Asian populations more commonly exhibit Th17- and neutrophil-dominant inflammation, highlighting the geographic and immunologic heterogeneity of CRSwNP [29, 34]. Beyond classical Th17 responses, emerging evidence supports immune plasticity within NP tissue. Recent studies have demonstrated that subsets of FOXP3⁺ T_regs_ can acquire IL-17A-producing capacity, particularly within CD4⁺CD45RA⁻FOXP3⁺ T_reg_ populations in CRSwNP. These IL-17A-secreting T_regs_ are significantly increased in polyp tissue and may contribute to persistent inflammation and tissue remodeling, linking impaired immune regulation to Th17-driven pathology [35]. These findings suggest that Th17-related pathways, together with T-cell plasticity and regulatory dysfunction, contribute to CRSwNP pathogenesis and may provide therapeutic targets in selected endotypes.

### Th9 Cells

Th9 cells are a distinct T-helper subset characterized by high IL-9 production. Increased IL-9 expression has been identified in sinonasal mucosa of CRS patients, particularly those with CRSwNP or eosinophilic CRS, compared with healthy controls [36]. IL-9 production can also be stimulated by Staphylococcus aureus and its enterotoxin B, and increased numbers of IL-9-producing neutrophils and mononuclear cells have been demonstrated in CRSwNP tissue [37]. Moreover, neutralization of IL-9 ameliorated symptoms of allergic rhinitis by reducing Th2, Th9, and Th17 responses and increasing the T_reg_ response in a murine model of allergic rhinitis [38]. These findings support a potential role for IL-9 in CRSwNP pathogenesis.

### Th22 Cells

Th22 cells are another Th subset implicated in the pathogenesis of allergic and autoimmune diseases. Th22 cells are primarily characterized by high secretion of IL-22, while producing relatively low levels of other cytokines such as IL-13 and TNF-α. IL-22 signaling occurs through the IL-22 receptor complex (IL-22R1/IL-10R2) and plays an important role in antimicrobial defense, inflammation, and epithelial tissue repair [39]. IL-22 can be produced by multiple immune cell types, including Th1, Th2, Th17, and Th22 cells, NK cells, and group 3 innate lymphoid cells [40, 41], whereas IL-22R1 expression is largely limited to epithelial cells rather than immune cells [42]. In the sinonasal mucosa, expression of IL-22 and IL-22R1 has been detected in both healthy controls and patients with CRSwNP, with IL-22 predominantly localized to infiltrating inflammatory cells and IL-22R1 mainly expressed by epithelial cells [43, 44]. IL-22R1 expression is significantly reduced in NPs compared with uncinate tissue from non-CRS subjects, particularly in patients with recalcitrant disease, suggesting dysregulated IL-22 signaling in CRSwNP [44]. Studies demonstrated that NP-derived cells produce IL-22 in response to Staphylococcus aureus exotoxins, and that IL-22 enhances mucin-1 (MUC1) mRNA expression in NPs. Dysregulated IL-22/IL-22R1 signaling has been associated with local eosinophilic inflammation, potentially through alterations in MUC1 expression [43]. Consistent with these findings, downregulation of MUC1 has been linked to corticosteroid resistance in patients with CRSwNP, suggesting the potential clinical relevance of IL-22–mediated epithelial regulation [45].

### Regulatory T-cell Dysfunction

**Fig. 1:**
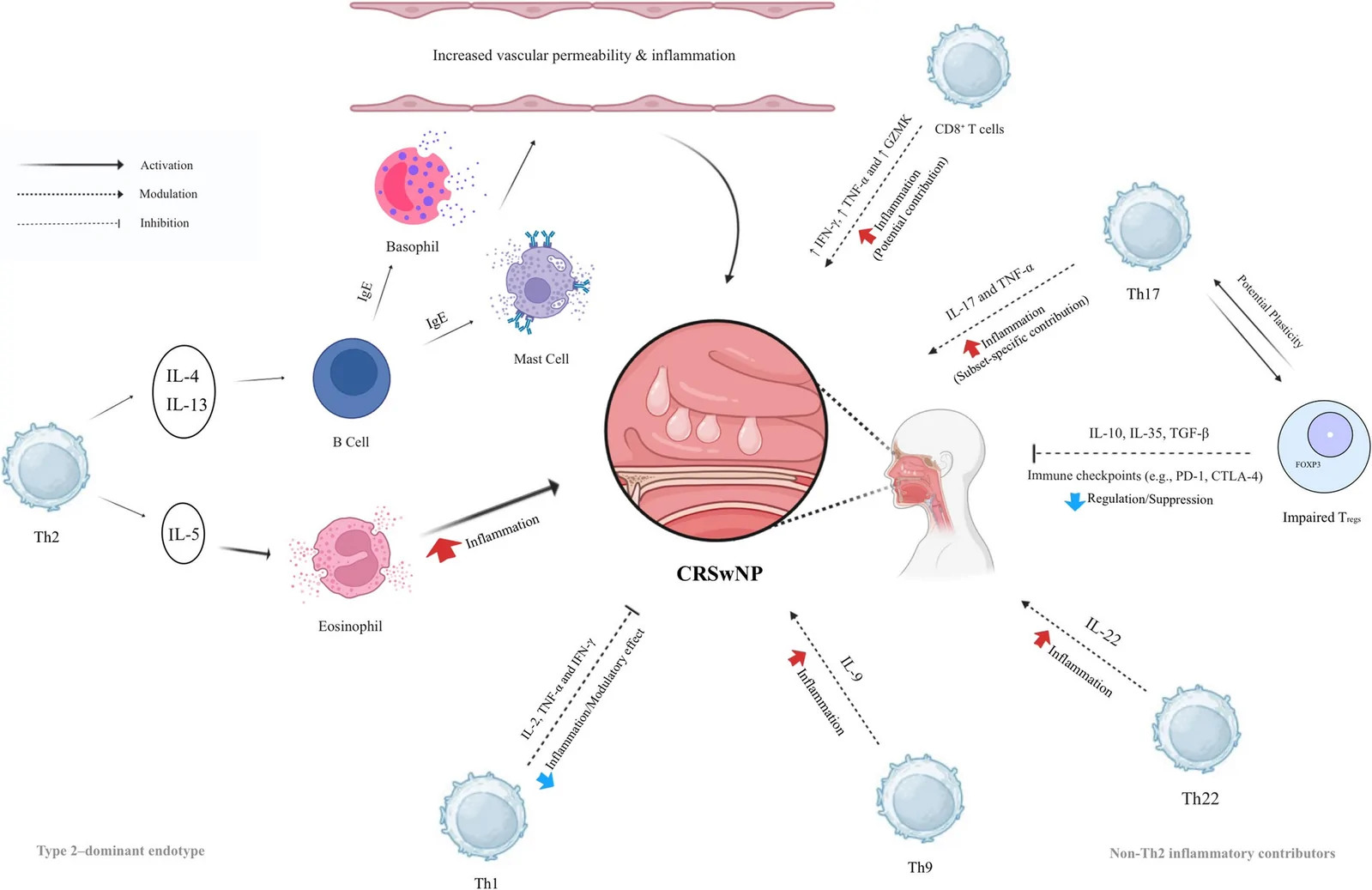
Core and emerging immunological mechanisms involving T helper cell subsets, CD8⁺ T-cells, and FOXP3⁺ regulatory T-cells (T_regs_) in CRSwNP. Within NP tissue, T-cells differentiate into multiple functional subsets that shape the local inflammatory microenvironment. The left side illustrates the predominant type 2 inflammatory axis, in which Th2-derived IL-4 and IL-13 promote IgE production and activation of mast cells and basophils, whereas IL-5 drives eosinophil recruitment and activation. The right side summarizes additional T-cell subsets that may contribute to inflammation in selected inflammatory endotypes, including Th17, Th9, Th22, and CD8⁺ T-cells. Th1-derived IL-2 is highlighted for its role in supporting T_reg_ survival and function. FOXP3⁺ T_regs_ normally suppress immune activation through regulatory cytokines (IL-10, IL-35, and TGF-β) and immune checkpoint pathways (PD-1 and CTLA-4), whereas impaired T_reg_ function contributes to persistent inflammation and immune imbalance in CRSwNP. Solid arrows indicate activating pathways, blunt-ended lines indicate inhibitory effects, and dashed arrows represent modulatory or context-dependent interactions

T_regs_ are essential for maintaining immune tolerance and limiting excessive inflammatory responses. The transcription factor forkhead box P3 (FOXP3) is critical for the development, stability, and suppressive function of conventional T_regs_ [46]. Several studies have reported reduced FOXP3 expression or impaired T_reg_ activity in CRSwNP mucosa [34, 47–50], although these findings appear to vary across inflammatory endotypes [34, 51]. Treatment with intranasal steroids has been associated with increased FOXP3 expression in NP tissue, suggesting that restoration of FOXP3-associated regulatory activity may contribute to the anti-inflammatory effects of corticosteroids [52]. Impaired IL-2 signaling may contribute directly to T_reg_ dysfunction in CRSwNP. Reduced expression of IL-2, the IL-2 receptor, and phosphorylated signal transducer and activator of transcription 5 has been demonstrated in NP tissue and was positively associated with FOXP3 expression and T_reg_ abundance [53]. These findings suggest that disruption of the IL-2-STAT5 pathway may impair T_reg_ differentiation and maintenance, thereby weakening immune regulation and promoting persistent inflammation. At the mechanistic level, impaired IL-2 and TGF-β signaling may disrupt T_reg_ differentiation and function [54–56], while altered IL-10 expression may further contribute to an imbalance between regulatory and effector T-cell responses [56]. In addition, inhibitory immune checkpoints, including programmed death-1 (PD-1) and cytotoxic T-lymphocyte-associated antigen-4 (CTLA-4), are expressed on T-cell subsets in NP tissue and may contribute to local immune regulation [57, 58]. TGF-β signaling is a key component of the NP microenvironment and is closely linked to immune regulation and tissue remodeling [56]. TGF-β promotes FOXP3 induction and T_reg_ differentiation and supports the maintenance of FOXP3 expression and suppressive function in peripheral T_regs_ [59, 60]. In NP tissue, TGF-β also promotes fibroblast activation, extracellular matrix deposition, and structural remodeling [56, 61–63]. TGF-β1 may therefore have a dual role in NPs by limiting inflammatory responses while simultaneously promoting tissue remodeling and cellular growth [61]. Studies have reported heterogeneous patterns of TGF-β expression in NP tissue. TGF-β1 expression has been identified within inflammatory cells of NP tissue [64], whereas reduced TGF-β1 signaling has been reported in CRSwNP [49, 56]. In addition, in a Japanese cohort, TGF-β1 expression was significantly higher in non-eosinophilic than eosinophilic CRSwNP [51]. These apparently divergent findings may reflect endotype-specific differences in inflammatory patterns, cellular composition, and tissue remodeling. Reduced TGF-β signaling in some CRSwNP endotypes may therefore contribute to impaired immune regulation within NP tissue [54]. TGF-β also regulates CD103 expression on T-cells in NP tissue, suggesting a role in T-cell retention and local inflammatory responses [65]. Moreover, CRSwNP exhibits immune plasticity, in which FOXP3⁺ cells may acquire inflammatory features, including IL-17 A production. FOXP3⁺IL-17⁺ T-cells have been identified in NP tissue, and elevated IL-17 A-secreting T_regs_ have also been detected in sinonasal tissues from patients with CRSwNP [35, 66]. In vitro, staphylococcal enterotoxin B promoted the development of FOXP3⁺ T-cells with an IL-17-producing or Th17-like phenotype, with IL-6 and TGF-β contributing to this process [55, 66, 67]. These findings suggest that inflammatory signals within the NP microenvironment may shift FOXP3⁺ cells toward a proinflammatory phenotype, potentially linking regulatory dysfunction to persistent inflammation and tissue remodeling. T_reg_-associated immune patterns also appear to differ across CRSwNP endotypes. In eosinophilic CRSwNP, lower IL-10 expression and increased IL-5 and IL-13 expression have been reported. In contrast, non-eosinophilic CRSwNP has demonstrated increased expression of FOXP3, IL-10, IFN-γ, IL-17A, and TGF-β1 [51]. These findings suggest reduced regulatory activity and increased Th2 cytokine responses in eosinophilic CRSwNP, whereas non-eosinophilic disease may exhibit increased T_reg_-associated, Th1, and Th17 responses. CD103⁺CD161⁺ T-cells have also been identified in CRSwNP tissue. These cells expressed tissue-resident markers, including CD103, CD69, and CD45RO, together with high levels of IL-17, IFN-γ, CTLA-4, and PD-1, but lower levels of FOXP3 and CD25. In recurrent CRSwNP, CD161⁺CD8⁺ T cells were reduced compared with primary disease, further indicating differences in the frequency and function of tissue T-cell populations according to disease phenotype and stage [57]. Collectively, impaired T_reg_ abundance or function, disruption of IL-2 and TGF-β signaling, and the acquisition of IL-17-producing phenotypes may alter the balance between regulatory and effector T-cell responses in CRSwNP [68]. In particular, dysregulation of the T_reg_–Th17 axis may contribute to persistent inflammation and tissue remodeling. The proposed roles of helper T-cell subsets and _Tregs_ in the pathogenesis of CRSwNP are summarized in Fig. 1.

### Therapeutic Approaches in CRSwNP

Current therapeutic strategies for CRSwNP primarily aim to control inflammation and reduce polyp burden. Corticosteroids remain the cornerstone of treatment in CRSwNP. Intranasal (topical) corticosteroids are first-line therapy due to their broad anti-inflammatory effects on innate and adaptive immune responses, while short courses of systemic corticosteroids may be used as adjunctive therapy in selected patients with severe disease or acute exacerbations [69–74]. More recently, targeted biologic therapies have emerged as effective treatment options for patients with severe or refractory CRSwNP characterized by type 2 inflammation. Dupilumab, a monoclonal antibody targeting the IL-4Rα subunit and inhibiting IL-4 and IL-13 signaling, has showed significant clinical benefit in patients with CRSwNP [75–80]. Mepolizumab, which targets IL-5, has demonstrated improvements in polyp burden, quality of life, and need for surgery [81–85]. Omalizumab, which targets IgE, has also demonstrated clinical efficacy in patients with CRSwNP, particularly those with comorbid asthma [86]. In addition, Tezepelumab, a monoclonal antibody targeting thymic stromal lymphopoietin and currently approved for severe asthma, demonstrated efficacy in the phase 3 WAYPOINT trial in CRSwNP [87]. A small immunophenotyping study suggested that dupilumab may reduce circulating Th2-cell proportions, increase relative Th1-cell proportions, and reduce PD-1 expression on T_regs_, without consistent changes in peripheral cytokine gene expression or production [88]. Biologic therapies have demonstrated substantial efficacy in selected patient populations and represent an important therapeutic option for severe or recurrent disease. However, their high cost and variable endotype responsiveness highlight the need for improved immunologic stratification to optimize patient selection and long-term outcomes [3, 89].

### Future Directions

Although substantial advances have been made in understanding the immunopathology of CRSwNP, the mechanisms responsible for impaired T_reg_ function and T-cell plasticity remain incompletely understood. Clarifying how IL-2 and TGF-β signaling regulate T_reg_ stability across different inflammatory endotypes may improve biomarker discovery and identify patients most likely to benefit from targeted therapies. Emerging single-cell and spatial transcriptomic approaches may further define the cellular interactions underlying persistent inflammation and disease recurrence.

## Conclusions

CRSwNP develops within a complex inflammatory microenvironment characterized by dysregulated interactions between innate and adaptive immune pathways. Among T-cell subsets, accumulating evidence supports central roles for Th2 cells, Th17 cells, CD8⁺ T-cells, and dysfunctional T_regs_ in chronic inflammation, tissue remodeling, and disease persistence. The balance between effector and T_reg_ subsets is central to immune homeostasis. While Th1 cells may provide anti-polyp effects, Th2 and Th17 responses intensify inflammation, and T_regs_ normally counteract these pathways. In CRSwNP, however, T_reg_ frequency and function are often impaired, partly due to abnormalities in IL-2, IL-10, and TGF-β signaling. These abnormalities may weaken immune tolerance and favor persistent activation of pro-inflammatory pathways. Although corticosteroids and biologics targeting Th2 cytokines provide meaningful clinical improvement, durable disease control remains challenging. Greater insight into T-cell plasticity and the cytokine networks that shape effector and regulatory responses may facilitate more precise immunologic stratification, targeted therapeutic strategies, and improved long-term outcomes for patients with CRSwNP.

## Key References

- Kato A, Peters AT, Stevens WW, et al. Endotypes of chronic rhinosinusitis: Relationships to disease phenotypes, pathogenesis, clinical findings, and treatment approaches. Allergy. 2022. https://doi.org/10.1111/all.15074
  - ○ This comprehensive review summarizes the major inflammatory endotypes of chronic rhinosinusitis and links molecular immune mechanisms with clinical phenotypes, biomarkers, and therapeutic approaches. It provides an important framework for understanding immune heterogeneity in CRSwNP.
- Lan F, Li J, Miao W, et al. GZMK-expressing CD8⁺ T-cells promote recurrent airway inflammatory diseases. Nature. 2025. https://doi.org/10.1038/s41586-024-08395-9
  - ○ This landmark study identifies GZMK⁺ effector-memory CD8⁺ T-cells as important contributors to persistent airway inflammation and disease recurrence, expanding current understanding of T-cell–mediated mechanisms beyond classical type 2 inflammation.
- Moon S, Rha MS. Revisiting T-cells in Chronic Rhinosinusitis. Allergy Asthma Immunology Research. 2024. https://doi.org/10.4168/aair.2024.16.6.585Moon S, Rha MS. Revisiting T-cells in Chronic Rhinosinusitis. Allergy Asthma Immunology Research. 2024. https://doi.org/10.4168/aair.2024.16.6.585.
  - ○ This review provides an updated overview of T-cell subsets, immune plasticity, and regulatory mechanisms in chronic rhinosinusitis, with particular emphasis on their translational and therapeutic implications.
- Fokkens WJ, Lund VJ, Hopkins C, et al. European Position Paper on Rhinosinusitis and Nasal Polyps 2020 (EPOS2020). DOI: https://doi.org/10.4193/Rhin20.600.
  - ○ EPOS2020 remains the principal international guideline for the diagnosis and management of chronic rhinosinusitis and provides the current clinical framework for disease classification, endotyping, and treatment selection.

## Data Availability

No datasets were generated or analysed during the current study.

## Abbreviations

NPs: nasal polyps
CRSwNP: chronic rhinosinusitis with nasal polyps
CRS: chronic rhinosinusitis
CRSsNP: chronic rhinosinusitis without nasal polyps
IgE: immunoglobulin E
Tregs: regulatory T-cells
IL: interleukin
IL-2R: interleukin-2 receptor
Th: T helper cell
NK: natural killer
IFN-γ: interferon-γ
TNF-α: tumor necrosis factor-α
TGF-β: transforming growth factor-β
FOXP3: forkhead box P3
PD-1: programmed death-1
CTLA-4: cytotoxic T-lymphocyte-associated antigen-4
GZMK: granzyme K
MUC1: mucin 1
T-bet: T-box transcription factor TBX21
